# Super Damping of Mechanical Vibrations

**DOI:** 10.1038/s41598-019-54343-3

**Published:** 2019-11-28

**Authors:** Ka Yan Au-Yeung, Brian Yang, Liang Sun, Kehang Bai, Z. Yang

**Affiliations:** Department of Physics, The Hong Kong University of Science and Technology, Clearwater Bay, Kowloon, Hong Kong The People’s Republic of China

**Keywords:** Acoustics, Condensed-matter physics

## Abstract

We report the phenomenon of coherent super decay (CSD), where a linear sum of the displacement of several damped oscillators can collectively decay much faster than the individual ones in the first stage, followed by stagnating ones after more than 97% of the energy has been dissipated. The parameters of the damped oscillators for CSD are determined by the process of response function decomposition, which is to use several slow decay response functions to approximate the response function of a fast decay resonator. Evidence established in experiments and in finite element numerical simulations not only strongly supported the numerical investigations, but also uncovered an unexplored region of the tuned mass damper (TMD) parameter space where TMD’s with total mass less than 0.2% of a stainless steel plate can damp its first resonance at 100 Hz up to a damping ratio of 4.6%. Our findings also shed light onto the intriguing underline relationships between complex functions with different singular points.

## Introduction

Damped vibrations are common phenomena in many physical systems, including merging black holes^1^ and neutron stars^2^. The classical vibrations can be described by a simple expression in time domain and in frequency domain^3^. Mitigating unwanted vibration in machinery, buildings, bridges, airplane, satellites, etc. is still a great challenge in engineering science despite of worldwide intensive efforts over the last century^4,5^. The distribution of vibration energy is mostly within the first few low frequency resonant modes of the structures. Since its invention nearly 100 years ago^6^, the toned mass dampers (TMD’s) or dynamic vibration absorbers (neutralizers) are particularly effective in suppressing vibrations with discrete frequencies, because their maximum effect is within a narrow band width around their individual working frequency, which can be designed to suit particular application needs. As a result, they have been widely used to specifically target at the first few resonant modes in many structures^7–33^. For comprehensive summary and reviews, see refs. ^7–10^. A TMD can be generically described by a damped harmonic oscillator^6^. The first theoretical investigation of the optimum TMD design was carried out in 1928^11,12^. Optimizations with generic TMD’s soon followed and are still being pursued today^11–29^. Most TMD’s are made of cantilevers or mass-spring that each weighs about 100 g or more for working frequency below 100 Hz^30–32^. The optimizations have been limited to the parameter space where the total mass of the TMD’s was a few percent of the primary structures, even though in some cases the TMD mass was up to 35% of the primary structures^27,28^. Damping the vibration in free bodies, such as satellites and airplanes, are even more challenging. While part of the vibration energy of a supported structure can be dissipated by the supporting bodies, i. e., the vibration energy of a building or bridge can partially be transmitted to the ground, or the vibration of a floor can be partially transmitted to the building, the vibration energy of a free body can only be dissipated internally. As a result, active control is the preferred choice for free bodies^33–42^.

In this paper we report solid experimental evidence and theoretical analysis of super damping of the first resonant mode at 100 Hz of a nearly free elastic body (a stainless steel plate about 6.24 kg in mass) brought by effective TMD’s with total mass less than 0.2% of the steel plate. Under an impulse excitation, the decay of the first resonance of the plate was nearly complete within a time scale of 100 ms, or about 10 periods of the oscillation. As the plate had negligible internal loss, almost all the impulse energy was eventually dissipated by the TMD’s. This shows that all the previous studies have missed an important parameter space of TMD’s with added mass about 50 times lighter than the conventional ones. Such phenomenon is explained with the concept of coherent super decay. A design strategy of the damper parameters based on response function decomposition is developed to achieve such super decay.

## Basics of Coherent Super Decay

We first present numerical investigations on the coherent super decay. Consider a group of five damped oscillators with their time dependent displacement {*x*_*n*_(*t*)}, *n* = 1, 2, 5 and the combined displacement *x*(*t*) in the form

1A $$x(t)=\mathop{\sum }\limits_{n=1}^{5}{x}_{n}(t)$$

where

1B $${x}_{n}(t)={A}_{n}{e}^{-{\omega }_{n}^{I}t}cos({\omega }_{n}^{R}t)$$

Here ${\omega }_{n}^{I}\equiv \frac{{\omega }_{n}}{2{Q}_{n}}$, ${\omega }_{n}^{R}\equiv {\omega }_{n}\sqrt{1-\frac{1}{{Q}_{n}^{2}}}$, *ω*_*n*_, *Q*_*n*_, and *A*_*n*_ are the intrinsic frequency, the quality factor, and the vibration amplitude of the *n*-th oscillator *ω*_*n*_(*t*), respectively, and *t* is the time. The imaginary part of the angular frequency of the *n*-th oscillator ${\omega }_{n}^{I}$ is the inverse of the decay time constant (DTC) of the damped oscillator. As a typical example for illustration, we choose *Q*_*n*_ = 20, ${\omega }_{n}=1+\delta {\omega }_{n}$, {$\delta {\omega }_{n}$} = {−0.09, −0.0389, −0.0048, 0.0267, 0.0792}, and {*A*_*n*_} = {0.0515, 0.1516, 0.358, 0.229, 0.0792}. The unit for the angular frequencies is Hz. For simplicity but without losing generality, the angular frequency *ω*_*n*_ is chosen to be around 1 Hz because the results at higher frequencies can readily be obtained by simply shrinking the time scale proportionally, i. e., the results at time = 1 second for *ω*_*n*_ = 1 Hz in the present case is the same as the one at time = 0.01 seconds for *ω*_*n*_ = 100 Hz. The choice of *Q*_*n*_ = 20 is made because it represents a modestly damped oscillator^3–5^. With these parameters the DTC of each oscillator is $\frac{1}{{\omega }_{n}^{I}}\approx 40\,s$. As a modest task we show below that with the purposely chosen values of {*A*_*n*_} and {*δω*_*n*_} listed above, the total displacement *x*(*t*) behaves like a single oscillator with ${\omega }_{0}\approx 1$ Hz, DTC close to 20 s, and therefore a Q-factor *Q*_0_ = 10, which happens to be close to the experimental results presented in the later part of the paper.

**Figure 1:**
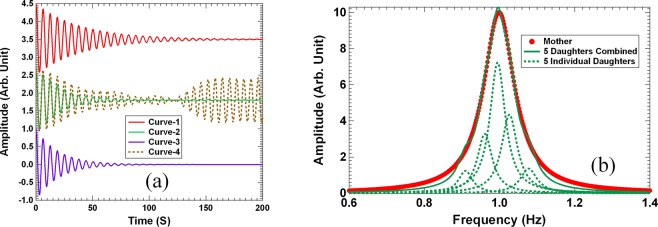
(**a**) The displacement as a function of time of several damped oscillators obtained by numerical computations. The curves are shifted vertically for clarity. (**b**) The reference response function (red disks) and the approximation response function (solid green curve) made by the sum of the response functions of five individual oscillators (green dashed curve) with the parameters given in the main text.

Figure 1(a) depicts the time dependent displacement curves for several vibration conditions. The initial amplitude of all the displacements is normalized to the value of 1. The curves are shifted vertically for clarity. The displacement of a representative oscillator *x*_3_(*t*) is shown in Fig. 1(a) as the red Curve-1. Its DTC is 40 s as determined by the resonant frequency and the Q-factor. As the Q-factors of the other four oscillators are also equal to 20, and their resonant frequencies deviate from *ω*_3_ by at most 9%, the DTC of each individual oscillator ranges from 44 s for the 1^st^ oscillator to 37 s for the 5^th^ oscillator, which are within ±10% of *x*_3_(*t*). Intuitively, one would expect the DTC of the combined displacement *x*(*t*) given by Eq. (1) to be about 40 s also. After all, if we set all {*δω*_*n*_} = 0, the combined displacement *x*(*t*) would almost exactly follow that of *x*_3_(*t*) regardless of the values of *A*_*n*_. However, the actual *x*(*t*) obtained is the green solid Curve-2 in Fig. 1(a). The DTC extracted from the curve is 20 s, which is about half of that of *x*_3_(*t*) or any of the other individual oscillators *x*_*n*_(*t*). In fact, the DTC is close to that of a single oscillator with *ω*_0_ = 1 Hz and *Q*_0_ = 10, the displacement of which is depicted as the purple Curve-3 in Fig. 1(a) for reference. In other words, when the parameters {*A*_n_} and {*δω*_*n*_} are properly selected as presented above, the combined decay of several oscillators *x*(*t*) can be much faster than each individual ones. We refer to such phenomenon as the *coherent super decay* (CSD) of harmonic oscillators.

The CSD is caused by the coherent interference among the oscillators. To see this more clearly, the time dependent combined displacement *x*(*t*) without the damping factors $\{{e}^{-{\omega }_{n}^{I}t}\}$ in Eq. (1) was also calculated and shown as the brown dashed Curve-4 in Fig. 1(a). It can be seen that the first minimum of the displacement by interference alone occurs at almost the same time when the combined decay (green solid curve) is near completion. At this moment, the phases of the individual oscillators are mutually destructive. Without damping, however, the displacement recovers its strength when the phases become mutually enhancing again as time goes by. With damping, sufficient vibration strength of each oscillator has already been consumed and the resurgence is negligible.

We now show an approach to determine the parameters that can realize CSD. According to classical mechanics^3^, the time dependent displacement given in Eq. (1) is resulted from the frequency response function given by

2A $$X(\omega )=\mathop{\sum }\limits_{n=1}^{5}\frac{{A}_{n}}{{\omega }_{n}^{2}-{\omega }^{2}+i\omega {\omega }_{n}/{Q}_{n}}$$

This is a typical frequency response function of a vibrating system with 5 coupled degrees of freedom, with

2B $${\tilde{\omega }}_{n}={\omega }_{n}\sqrt{1-\frac{1}{{Q}_{n}^{2}}}+i\frac{{\omega }_{n}}{2{Q}_{n}}$$

being the complex eigen-frequency of the *n*-th eigenmode, and the projection amplitude of the excitation on the *n*-th mode being *A*_*n*_^3,41^. The time dependent displacement in response to a unit impulse *δ*(*t*) is given by

3 $$x(t)=\mathrm{Re}(\frac{1}{2\pi }{\int }_{-\infty }^{\infty }X(\omega ){e}^{i\omega t}d\omega )$$

The integral can be calculated by using the standard path integral and residue theorem in complex analysis^3,41,42^ to recover Eq. (1). As the CSD behaves like a reference oscillator with resonant frequency = 1 and *Q*_0_ = 10, we plot the imaginary part of the response function of the reference oscillator in the form ${X}_{0}(\omega )=\frac{1}{{\omega }_{0}^{2}-{\omega }^{2}+i\omega {\omega }_{0}/{Q}_{0}}$ in Fig. 1(b) as the red disks. The real part is not shown due to space limit. The green solid curve is the imaginary part of the response function in Eq. (2) using the CSD parameters for Eq. (1). It is no coincidence that the two response functions are nearly the same, because we had deliberately chosen the amplitudes {*A*_*n*_} and the resonant frequencies {*δω*_*n*_} to approximate, or mimic, the reference oscillator response function with *Q*_0_ = 10. The green dashed curves are the response functions of the five mimicking resonators plotted individually, each with *Q* = 20. We refer to such process as *response function decomposition* (RFD), that is, if a response function with large line width can be mimicked by the sum of a number of response functions with much smaller line width, then the time dependent displacement generated by the two response functions will be almost the same, even though each mimicking oscillator has narrower line width (larger Q-factor) and decays much slower. That is the underline strategy to identify the amplitude and the central frequency parameters for the response functions with slow individual decay to collectively generate a much faster decay. In principle, the amplitude *A*_*n*_ in each term in Eq. (2) can be complex, which is equivalent to introducing an initial phase for each term in Eq. (1), as long as the final outcome via Eq. (2A) mimics the reference oscillator well. In this work we limit our search of *A*_*n*_ to real numbers just to avoid excessive numerical complications.

**Figure 2:**
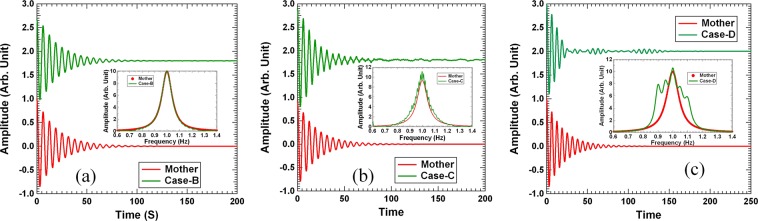
(**a**) The computed time dependent displacement of case-B (green curve) together with the reference (red curve). The insert depicts the response function of case-B (green curve) and that of the reference (red curve). (**b**) The corresponding quantities for case-C. (**c**) The corresponding quantities for case-D. All the time dependent displacement curves are vertically shifted for clarity. Their initial amplitudes are normalized to 1.

In the above example, we decompose one reference (mother) resonator of *Q*_0_ = 10 into five *Q* = 20 daughter resonators (Case-A). The same can be done to decompose each daughter resonator into five granddaughters with *Q* = 40, so as to mimic the mother resonator with 25 granddaughter resonators, each with four times the Q-factor of the mother. The resulting time dependent displacement curve is shown in Fig. 2(a), together with the response function in the insert (Case-B). The corresponding results by 125 great granddaughter response functions with *Q* = 80 (Case-C) are shown in Fig. 2(b). In Case-C we deliberately kept the mimicking curve relatively rough while keeping the average line shape nearly tracing the original mother resonator. Despite the rough mimicking, the time dependent displacement curve of the 125 great granddaughters is still very close to that of the mother resonator.

If the reference oscillator response function is poorly mimicked (Case-D), as is shown in the insert of Fig. 2(c), the resulting decay will resurge after initial fast decay, as is shown in the main graph, and the decay lasts for quite a long time.

For more in-depth examination of the decay behavior, we calculate the quantity that is proportional to the remaining total energy of a vibrating elastic body, given by

4 $$E(t)={\int }_{t}^{\infty }|x(t^{\prime} ){|}^{2}dt^{\prime} $$

**Figure 3:**
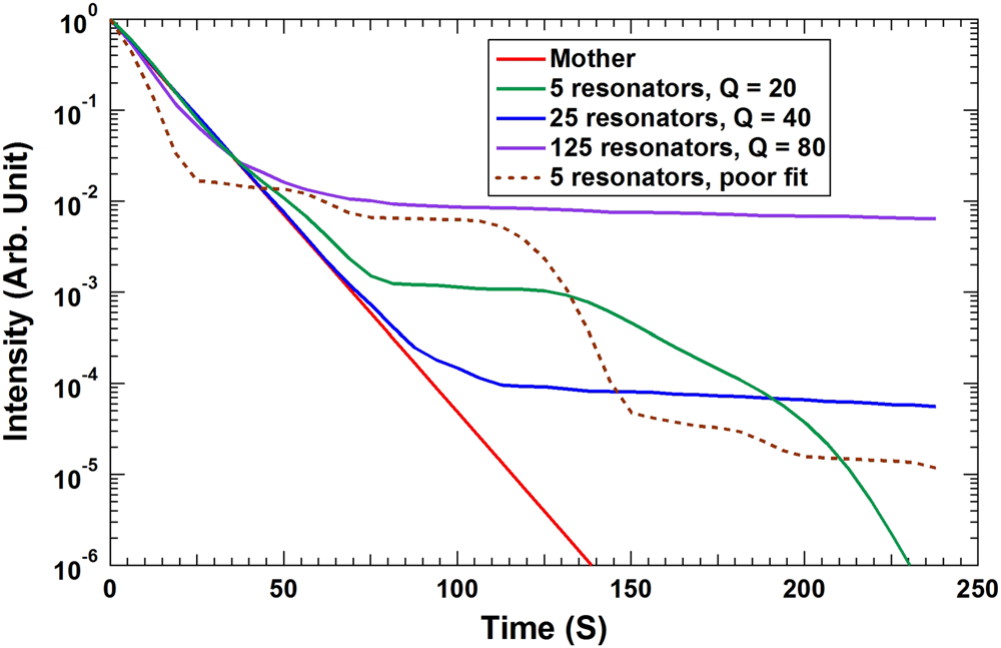
The remaining vibration intensity as a function of time for the cases studied above, together with that of the reference (red line).

The curve by the mother (reference) resonator is shown in Fig. 3 as the red curve, which takes the simple form of ${e}^{-0.1t}$. We refer to such decay as a uniform decay, in that the DTC remains the same throughout the decay process. The corresponding curve by the five daughters (Case-A) is shown as the green curve in the same figure, together with the one made by the 25 granddaughters (Case-B, blue curve) and the 125 great granddaughter resonators (Case-C, purple curve). All of them exhibit similar decay in the first stage as the reference, and staged decays with different DTC’s in different stages afterwards. This is the consequence that their mimicking of the reference resonator is only approximate.

For Case-A, the normalized decay curve in the first stage decay follows closely that of the reference resonator up to *t* = 38 s (6.05 periods for an angular frequency of 1 Hz) where *E* = 2.56%. The initial quick drop levels off at around *t* = 81 s, after which the system is in the stagnating second decay stage. However, by then only 0.12% of the original vibration energy still remains, so in practical applications the stagnation will not have any material implications in the vibration damping of a primary structure. The reference resonator will take 69 s to reach such energy reduction. Therefore, for all practical engineering purposes Case-A can be regarded as decaying as fast as the reference.

The decay curve of Case-B (blue curve) matches well with the reference up to *t* = 69 s, where *E* = 0.1%. The curve for Case-C (purple curve) decays even slightly faster than the reference up to *t* = 38 s, where *E* = 2.5%. This is consistent with its response function being a little wider in the combined line width than the reference. It levels off at *t* = 69 s, with *E* = 1.0%.

Case-D with poorly mimicking response function is also interesting. Its apparent line width is almost twice of that of the reference. So the question is when it will slow down after the initial fast decay. The result, shown as the brawn dashed curve in Fig. 3, indicates that it takes only *t* = 25 s (4 periods) to bring *E* down to 2.0%, as compared to 44 s for the reference. At the time of leveling off of the decay curve, only 1.7% of the initial energy is still present. Therefore, in practical applications, the decay can already be regarded as complete, and the damper parameters chosen to create such response function are really good for optimization purposes.

### Experimental verifications

The CSD realized by following the RFD strategy can lead to the design and realization of fast vibration decay of primary structures brought by multiple TMD’s that are much lighter than the ones reported in the literature^4–29^, as will be shown in the experiments below.

**Figure 4:**
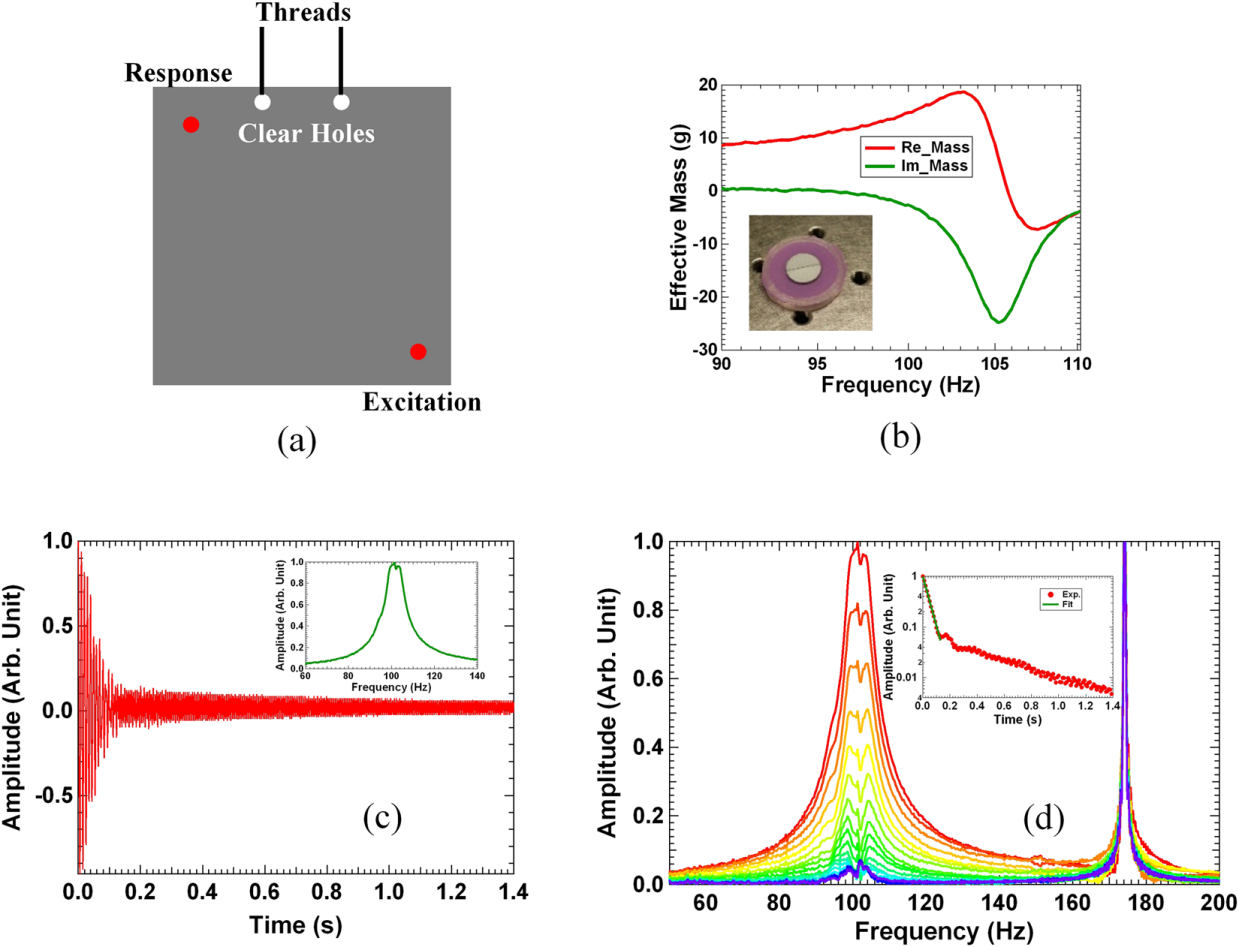
(**a**) The schematic of the experimental setup. The red dot at the lower right corner is the location of excitation, while that on the upper left corner is the location where the response displacement is measured. (**b**) The real part (red curve) and the imaginary part (green curve) of the experimental dynamic mass of a typical DMR damper. The insert is a photo of a DMR damper. (**c**) The normalized experimental time trace of the displacement of the steel plate with the four DMR dampers attached after a single impulse excitation. The insert depicts the experimental response function. (**d**) The delayed Fourier transform spectra at different delay time obtained from the time trace in (**c**). The insert is the time dependent peak amplitude (red disks) obtained from the spectra and the exponent fitting (the green line).

The primary structure for the damping experiments is a 40 cm × 40 cm × 5 mm stainless steel plate (6.24 kg in mass) with free edges. Figure 4(a) is a schematic of the experiment setup. The steel plate was hanging by soft threads through the two clear holes near its top edge at about 10 cm apart. For frequency response measurements, the plate at the position 7 cm from the lower right corner along the plate diagonal line was attached to a shaker similar to the one reported earlier^43^. The response displacement was measured by a miniature accelerometer at the corresponding position relative to the left upper corner. Single frequency excitation was used, and the excitation and the response displacement signals were measured by lock-in amplifiers. The first eigenmode of the bare steel plate was found to be 100.24 Hz with a Q-factor over 3000 (damping ratio *η* = 0.5/*Q* = 1.7 × 10^−4^). This is expected for a bare stainless steel plate free body. For free vibration decay measurements, the shaker was removed, and a gentle knock was applied near the same position where the shaker was originally attached, and the time dependent displacement of the plate vibration was recorded by a data collection card afterwards.

For an elastic body such as the steel plate here, the response function of the vibration in a particular direction (the off-plane direction of the plate in this case) is given by the Green’s function formulism^41,44^,

5 $$G(x,x^{\prime} )=\mathop{\sum }\limits_{n=1}^{\infty }\frac{{u}_{n}(x){u}_{n}^{\ast }(x^{\prime} )}{{m}_{n}({\omega }_{n}^{2}-{\omega }^{2}+i\omega {\omega }_{n}/{Q}_{n})}$$

where *u*_*n*_(*x*) is the vibration field of the *n*-th eigenmode, *x* is the position where the response displacement is measured, *x*’ is the position of excitation, ${m}_{n}\equiv \int \rho (\overrightarrow{r}){u}_{n}^{2}(\overrightarrow{r})dv$, and $\rho (\overrightarrow{r})$ is the mass density distribution of the elastic body. For a bare steel plate the frequencies of the first few lowest primary eigenmodes are well separated. For example, the frequency of the 2^nd^ primary eigenmode is near 125 Hz. When four miniature TMD’s with their working frequencies strategically selected around the 1^st^ eigen-frequency of 100 Hz are mounted on the plate, five secondary eigenmodes near the original primary eigenmode at 100 Hz are generated. The time dependent vibration displacement of any part of the object would be of the form as Eq. (1) with each oscillator corresponding to an eigenmode, and the summation includes all the eigenmodes, including the primary and the secondary ones^3^. The response function near 100 Hz would be reduced to the same form as Eq. (2A) if the higher primary eigen-modes are only weakly excited and be neglected. As has been mentioned above, the results obtained in the numerical studies of CSD done at angular frequency of 1 Hz can be directly applied here for the angular frequency of 628 Hz by simply shrinking the time scale by a factor of 628. The dampers are purposely fine-tuned to collectively produce a response function that mimics a single peak around 100 Hz with an apparently wider line width and smaller effective Q-factor. It was then hoped that, if the numerical studies are correct, CSD would occur in the real mechanical system and be verified experimentally.

The miniature TMD’s used in this study were made of decorated membrane resonators (DMR’s) reported earlier^43,45^. The photo of one of the DMR’s used in the experiments is shown in the insert of Fig. 4(b). The diameter of each stretched membrane was 25 mm with its boundary fixed on the circular plastic frame about 5 mm in width and 5 mm in height. The thickness of the rubber membrane is 0.2 mm. The frame weighs about 0.5 g. A steel platelet 10 mm in diameter and 1.0 g in mass was attached to the center of the membrane. The total mass of a DMR-type TMD is therefore about 1.5 g, while the oscillating mass is about 1.0 g. The mass density, Poisson’s ratio, Young’s modulus, and pre-stress of the membrane were 980 kg/m^3^, 0.49, 5 × 10^5^, and 0.4 MPa, respectively. The pre-stress was estimated by matching the theoretical eigen-frequency obtained from simulations to the experimental one near 100 Hz. The method to measure the dynamic effective mass of the DMR’s reported earlier^43^ was used here. In simple terms, the dynamic mass of the DMR was obtained by the measured force acting on the frame divided by the acceleration of the frame, with the frame of the DMR firmly mounted on the shaker. Vacuum grease was applied on the membrane to reduce its Q-factor from around 100 for a pristine DMR to about 25. The second resonance of the DMR’s was above 500 Hz so it will not be considered further in this work. Small amount of plasticine was added onto the platelet to fine tune the first resonance frequency of each DMR, so that collectively they would spread around the first primary resonance of the steel plate near 100 Hz. Due to the very small mass of the miniature TMD’s, the resonance peak of the plate only shifted by about 0.3 Hz when 4 DMR’s were attached, one to each of the four corners of the steel plate.

A typical measured dynamic mass of a DMR is shown in Fig. 4(b). We chose the convention of negative imaginary mass for energy dissipation. The positive imaginary part of the eigen-frequency would then lead to the decay of vibration amplitude with time. At the first resonance of the DMR, the maximum amplitude of the imaginary mass was about 25 g, while the Q-factor obtained from the line width at half height is about 25. It is noted that for a classical TMD, the peak imaginary mass *M*_*i*_ is equal to the Q-factor times the oscillator mass *M*_0_, i.e., *M*_*i*_ = *QM*_0_, which is well validated here. It is a clear indication that the DMR behaves like a classical TMD as far as its damping effect on a primary structure is concerned. Also, the line shapes of the real and the imaginary parts of the dynamic mass resemble well the Lorentzian form that satisfies the Kramers-Kronig relations.

The measured first resonant frequency of each of the four DMR’s is listed in the first row in Table 1. These frequencies were the results of fine-tuning so that with these DMR’s, one at each corner of the steel plate, the resulting frequency response of the steel plate near 100 Hz is shown in the insert of Fig. 4(c). The reason to place the DMR’s at the corners of the steel plate is that the vibration amplitude of the first resonance of the plate is the largest at the corners. The line shape of the frequency response nearly resembles a single resonance with an apparent Q-factor of 13 as estimated from the line width, even though the total mass of the dampers is less than 0.1% of the primary structure, which is in stark contrast to the typical ones in the literature which are more than 10 to 100 times heavier^27,28,30–32^. As a reminder, it is noted that the Q-factor of the bare steel plate is close to 3000. The decrease of the Q-factor is quite impressive. However, the question of whether such small value of the apparent Q-factor would really bring fast vibration decay in the free vibration of the steel plate still remains to be answered. Compared to the added mass by multiple TMD’s of the order of 5% or more of the primary structure reported in the literature, our dampers would have been considered as insignificant, let alone causing significant damping. We have also noticed that the measured frequency response remained almost unchanged when the damper on the upper left corner was moved to the upper right corner, such that there was no damper on the upper left corner while there were two on the upper right corner.

**Table 1:** The measured peak frequency of the imaginary effective dynamic mass of each DMR damper used in the experiments.

Damper #	#1	#2	#3	#4	#5	#6	#7	#8
Group-1	99.3	99.3	105.2	105.3				
Group-2	96.2	98.2	100.4	101.4	101.6	101.8	102.8	104.2

The question of whether such frequency response with an ‘apparent’ Q-factor of 13 would indeed produce a fast decay comparable to such Q-factor is answered by the normalized experimental time dependent displacement curve shown in Fig. 4(c). Fast decay of vibration is indeed observed within the first 70 ms (~7 periods) after an impulse excitation. The remaining slow decay is mostly due to the second primary resonance of the plate around 125 Hz. To examine the displacement curve more closely, we carried out Fourier transform of the remaining portion of the time-dependent curve after certain delayed time from the impulse, i. e, delayed Fourier transform analysis. The selected moments of time were the ones at the consecutive maximum positive displacement where the phase is an integer number of 2*π* to avoid oscillatory behavior in the amplitude decay curve obtained from the delayed Fourier transform. Each spectrum was therefore taken at about 10 ms (~1 period) later than the earlier one. The resulting spectra are shown in Fig. 4(d), where a fast decrease of the peak amplitude around the first resonance of 100 Hz of the steel plate can be clearly seen. In the insert of Fig. 4(d) is the normalized amplitude of the peak versus the delay time (the red disks). A fast decay in the first stage followed by a second stage slow decay is clearly seen. A DTC of 44 ms is extracted from fitting the first stage decay curve with an exponent function (the green line). The corresponding Q-factor is 14, which is in good agreement with the one estimated from the response function of the steel plate in the insert of Fig. 4(c), verifying experimentally the mutual dependence of the time dependent vibrational displacement in the time domain and the corresponding response function in the frequency domain. The second stage decay was also clearly revealed, with its DTC much larger than that of the first stage. The 2^nd^ eigenmode at 125 Hz was also excited, but within the decay time scale of the 1^st^ eigenmode it decayed little, as expected.

**Figure 5:**
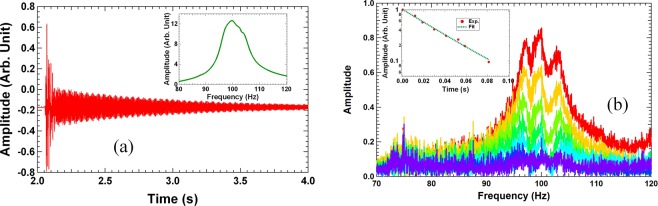
(**a**) The experimental time trace of the displacement of the primary structure with 8 DMR dampers attached. The insert depicts the experimental response function. (**b**) The delayed Fourier transform spectra at different delay time obtained from the time trace in (**a**). The insert is the time dependent peak amplitude obtained from the spectra.

To further increase the damping effect, four more DMR’s were added, one at each corner, and their resonant frequencies were fine-tuned to obtain the experimental response function as shown in the insert of Fig. 5(a), which nearly resembles a single oscillator with an apparent *Q* = 11. The time trace is shown in Fig. 5(a). The resonant frequencies of the eight dampers are listed in the second row in Table 1. The delayed Fourier transform spectra of the decay curve are shown in Fig. 5(b), while the peak amplitude decay curve is shown as the insert in the same figure. The obtained Q-factor is 10.8, which again matches well with the one from the measured response function. The decay time constant is 35 ms. It has also demonstrated the damping efficiency of the DMR’s with total mass of 12 g, or less than 0.2% of the primary structure, that can bring out a damping ratio as high as 4.6% for the first resonance of the steel plate.

Although in principle the response function, and therefore the decay behavior, depends on the locations of excitation and response, our experimental investigations showed that the super decay phenomenon was quite robust. Applying the impact excitation at different locations of the steel plate resulted in the variation of relative strength of the eigenmodes being excited, but as long as the 1^st^ primary eigenmode is excited, its decay followed the same as reported above, so the experimental findings presented above are robust.

### Simulations

**Figure 6:**
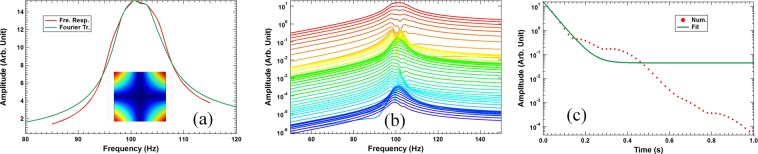
(**a**) The frequency response functions of the primary structure with four attached DMR dampers obtained directly from simulations (the red curve), and from mimicking using the five secondary eigenmodes (the green curve). The insert shows the vibration pattern of the first resonance of the bare steel plate. (**b**) The delayed Fourier transform spectra obtained from the time dependent displacement obtained from simulations. (**c**) The amplitude of the peak in (**b**) as a function of delay time (the red dashed curve) and the numerical fit by a single exponent function with a small offset (the green curve).

The finite element simulations for the damping of the primary structure by the four DMR dampers in the experimental investigation were performed by using COMSOL multi-physics using the following materials parameters for the steel plate, namely density = 7850 kg/m^3^, Young’s modulus = 2.0 × 10^11^ Pa, and Poisson ratio = 0.33. The vibration pattern of the first primary resonance of the bare steel plate is shown in the insert of Fig. 6(a). The red color indicates high vibration amplitude, while the deep blue color indicates near zero vibration. For damping simulations, instead of calculating the steel plate with the real DMR’s, which could take up a large amount of computer memory and computational power because of the thin membrane structure of the DMR’s, the measured dynamic mass spectra of the DMR’s were used instead as the effective input parameters for their damping effects^43^. The dampers were placed at the corners of the plate because it is where the vibration amplitude of the first resonance is the largest.

The numerical simulations were carried out in the following steps. We first compare the response function, and then the characteristics of the time dependent displacement with the experiments. In doing so, we also verify theoretically the mutual dependence of the time dependent vibrational displacement of a real object in the time domain and the corresponding response function in the frequency domain.

In step-1, the response function of the primary structure in the same excitation-response scheme as the experiments was calculated when four DMR dampers with the parameters shown in Table 1 were attached to the steel plate. The resulting response function is shown as the red curve in Fig. 6(a), with an apparent *Q* = 11, which agrees well with the experimental one shown in the insert of Fig. 5(a) with *Q* = 13. The reason for the small discrepancy in the Q-factor is most likely due to the imperfection of the real DMR dampers. The numerical response function cannot provide time dependent vibration of the steel plate to compare with the experimental results. According to Eq. (1), doing so requires the numerical eigen-frequencies {${\tilde{\omega }}_{n}$}, which will be obtained in step-2, and the corresponding mode amplitudes {*A*_*n*_} of the steel plate, which will be obtained in step-3 below.

In step-2 the lowest eigen-frequencies of the steel plate with the four dampers attached onto the corners were calculated. It was found that the original primary mode near 100 Hz of the bare steel plate now splits into five secondary modes, because four additional virtual degrees of freedom were introduced by the four effective TMD’s. The eigen-frequencies of the secondary modes obtained from the simulations are $\{\frac{{\tilde{\omega }}_{n}}{2\pi }\}$ = {96.4678 + 1.74668*i*, 98.2078 + 2.06091*i*, 100.159 + 1.80042*i*, 102.455 + 2.4188*i*, 104.085 + 2.02823*i*} in Hz, revealing the induced secondary modes with the frequencies spreading across the original primary resonance of the steel plate and the imaginary parts due to DMR damping. The complex eigen-frequency of the *n*-th secondary eigenmode ${\tilde{\omega }}_{n}$ is related to *ω*_*n*_ and *Q*_*n*_ via Eq. 2(B). Accordingly, the Q-factors of these secondary modes, given by the ratio of the real part of the eigen-frequency over the imaginary part, range from 55 to 43, none being close to the experimental value of 13, or the apparent Q-factor of 11 in the simulations. Therefore, the response function obtained in step-1 is a ‘mimicked’ one by the combination of the individual response functions of these secondary modes with a factor of 3 to 4 times narrower line widths. As the imaginary part of the eigen-frequencies is much smaller than the real part, the eigen-mode vibration field (*u*_*n*_(*x*) in Eq. (4)) is mostly real, leading to negligible initial phase in each oscillator. Our choice to exclude initial phases in Eq. (1) is therefore well justified.

In step-3, the response function amplitudes of the five secondary modes, which corresponds to the {*A*_*n*_} in Eq. (1), were then found following Eq. (2) to produce a response function that mimics the one obtained in step-1. The resulting response function is shown as the green curve in Fig. 6(a), which matches well with the red one obtained in step-1.

With all the parameters to calculate *x*(*t*) using Eq. (1A) now available, in step-4 the time dependent displacement was then calculated, followed by the delayed Fourier transform on the numerical *x*(*t*). The resulting spectra are shown in Fig. 6(b). The time interval between two consecutive spectra is about 20 ms, or 2 periods. The evolution of the spectra resembles well the experimental one in Fig. 4(d).

Finally, in step-5 the total energy decay curve was calculated by using Eq. (4) and the time dependent displacement found in step-4. The outcome is shown in Fig. 6(c). The decay curve clearly exhibits the characteristic multi-stage fast and slow decays similar to that in Fig. 3. The decay started with a fast drop that lasts for about 100 ms, followed by a slow decay till about 200 ms, then fast decay again. The Q-factor obtained from the first stage fast decay is 12, which is in good agreement with the value of 11 obtained from the spectrum in Fig. 6(a), and the experimental value of 14. The DTC so obtained is 35 ms, which agrees fairly well with the experimental value of 44 ms. The decay in the remaining stages is negligible because the vibration amplitude is already minute.

Overall, the results from the simulations agree well with the experimental ones both in terms of the frequency response function and in terms of DTC. Furthermore, the mutual dependence of the time dependent vibrational displacement in the time domain and the corresponding response function in the frequency domain has been firmly verified both experimentally and theoretically.

## Discussions

Although we have only reported the damping of the first resonance of the primary structure, it is straightforward to damp the second resonance which has maximum vibration at the center of each edge, away from that of the first resonance. One would expect that four dampers similar to the one in Fig. 2(a) are needed. The total added mass to damp the first two resonances would most likely be below 0.5%. As for most structures the vibration energy is concentrated in the first few resonances, our approach could suppress the vibration of nearly free bodies with no more than 1% added mass by TMD’s.

After nearly 100 years since the invention of TMD’s, the parameter space for optimizing the performance of TMD’s seem to have already been exhaustively combed. What we report in this work reveals a piece of fertile and yet unexplored land in the parameter space, where TMD’s with mass only a fraction of a percent of the primary structure can effectively tame the individual resonant modes of the primary structure. The intuitive method of using the apparent line width in the frequency response function to adjust the design parameters of the TMD’s for effective damping is simple to use in designs and in experiments. The non-uniform multiple-stage decay processes where the decay time constant changes in different stages, such that at the beginning there could be a fast decay of most vibration energy followed by a stage of stagnating decay, could be very beneficial for practical applications, where the real effect of the remaining few percent of the initial vibration energy on the primary structure after a stage-1 rapid decay is almost negligible. Therefore, the effectiveness of the dampers is almost entirely determined by the first stage.

Our findings also reveal some interesting underline connections between two complex functions which are mutual approximation of each other. Take for example the case of the mother resonance ${X}_{M}(\omega )=\frac{1}{{\omega }_{0}^{2}-{\omega }^{2}+i\omega {\omega }_{0}/{Q}_{0}}$ being approximated by five daughter resonances ${X}_{D}(\omega )=\mathop{\sum }\limits_{n=1}^{5}\frac{{A}_{n}}{{\omega }_{n}^{2}-{\omega }^{2}+i\omega {\omega }_{n}/{Q}_{n}}$. *X*_*M*_(*ω*) and *X*_*D*_(*ω*) are a mimicking pair, that is, $\mathrm{Re}({X}_{M})\approx \mathrm{Re}({X}_{D})$ and $\text{Im}({X}_{M})\approx \text{Im}({X}_{D})$, and are expressed in the form of ${X}_{M}(\omega )\triangleq {X}_{D}(\omega )$ to denote such relation. Furthermore, ${x}_{M}(t)\approx {x}_{D}(t)$, where5Aand5B

**Figure 7:**
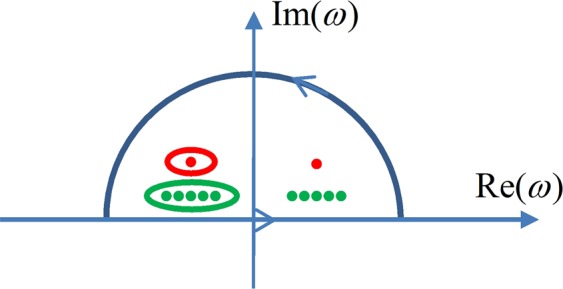
The path (semicircle) to carry out the path integral in Eq. (5), and the simple poles (red and green dots).

The closed integral path is an infinitely large semicircle shown in Fig. 7, with its straight edge along the real axis of the complex *ω*- plane^42^.

The expression ${x}_{M}(t)\approx {x}_{D}(t)$ means that the two functions are nearly equal in the first stage, as exemplified in Fig. 3 and in Fig. 6(c), and the difference between them is negligible when compared to their initial strength. However, the two complex functions cannot be equal in the domain of complex analysis. The complex function *X*_*M*_(*ω*) has two simple poles with residue ‘1’ in the complex *ω*-plane, as represented by the two red dots in Fig. 7. The complex function *X*_*D*_(*ω*), on the other hand, has ten simple poles (the green dots in Fig. 7) with proper residues, both taking the values according to the mimicking conditions given in Eq. (1). The poles are closer to the real axis because they have smaller imaginary parts as compared to *X*_*M*_(*ω*). If one carries out the path integral along the red loop in Fig. 7, the integral with *X*_*D*_(*ω*) will be zero while that with *X*_*M*_(*ω*) will be of non-zero. Likewise, along the green loop the path integral with *X*_*M*_(*ω*) will be zero while that with *X*_*D*_(*ω*) is non-zero. In more general terms, for two complex functions *F*_1_(*ω*) and *F*_2_(*ω*), ${F}_{1}(\omega )\triangleq {F}_{2}(\omega )$ when *F*_1_(*ω*) only has two simple poles as *X*_*M*_(*ω*) with the same residues, and *F*_2_(*ω*) only has ten simple poles and the residues as those in *X*_*D*_(*ω*), as long as the integrals along the big semicircle is negligible for both *F*_1_(*ω*) and *F*_2_(*ω*). As any response function can be mimicked by almost an infinite number of combinations of mimicking functions with their own simple poles and residues, a complex function with two simple poles would somehow be related to many other complex functions with the right poles and residues. Such intriguing mathematics is yet to be explored.
